# Spatial analysis of food insecurity and obesity by area-level deprivation in children in early years settings in England

**DOI:** 10.1016/j.sste.2017.07.001

**Published:** 2017-11

**Authors:** Sara E Benjamin Neelon, Thomas Burgoine, John A Gallis, Pablo Monsivais

**Affiliations:** a UKCRC Centre for Diet and Activity Research (CEDAR), MRC Epidemiology Unit, University of Cambridge School of Clinical Medicine, Box 285, Institute of Metabolic Science, Cambridge Biomedical Campus, Cambridge CB2 0QQ, UK; b Department of Health, Behavior and Society, Johns Hopkins Bloomberg School of Public Health, 624 North Broadway, Baltimore, MD, USA; c Department of Biostatistics and Bioinformatics, Duke University Medical Center, Durham, NC, USA

**Keywords:** Area-level deprivation, Food insecurity, Obesity, AIC, akaike information criterion, CI, confidence interval, GIS, geographic information system, GWR, geographically weighted regression, IMD, index of multiple deprivation, LSOAs, lower super output areas, OR, odds ratios, UK, United Kingdom, US, United States

## Abstract

**Background:**

we assessed manager perceptions of food security and obesity in young children attending nurseries across England, assessing spatial differences by area-level deprivation.

**Methods:**

we conducted an adjusted multinomial logistic regression and an adjusted geographically weighted logistic regression examining the odds of a manager perceiving obesity, food insecurity, or both as a problem among children in care measured via a mailed survey.

**Results:**

851 (54.3%) managers returned the survey. A nursery being in the highest tertile of area-level deprivation was associated with a 1.89 (95% CI 1.00, 3.57) greater odds of perceiving obesity as a problem, a 3.06 (95% CI 1.94, 4.84) greater odds of perceiving food insecurity as a problem, and a 8.39 (95% CI 4.36, 16.15) greater odds of perceiving both as a problem, compared with the lowest tertile.

**Conclusions:**

we observed differences in manager perception by area-level deprivation, but the relationship was especially pronounced for food insecurity.

## 1 Introduction

Obesity is associated with numerous adverse health and behavioral conditions—even in early childhood (Juonala et al., 2011, Kuhl et al., 2012; Llewellyn et al., 2016; Puder and Munsch, 2010, Simmonds et al., 2016). Thus, the prevention of childhood obesity is a public health priority in the United Kingdom (UK). Although rates of obesity in early childhood have shown some improvement in recent years, over 20% of children aged 2–4 years are currently overweight or obese (National Child Measurement Programme England, 2015–2016 School Year, Health and Social Care Information Centre). Moreover, as in adults, there are persistent social inequalities in the prevalence of obesity (Anderson and Whitaker, 2009, Ogden et al., 2015, Zilanawala et al., 2015). These inequalities have widened in recent years, with children from the lowest socioeconomic groups showing the sharpest rises in obesity (National Child Measurement Programme England, 2015–2016 School Year, Health and Social Care Information Centre).

At the same time, food insecurity, also known as food poverty, has emerged as an important social and public health concern in England (Too Poor to Eat, Food Insecurity in the UK). Food insecurity, characterized as limited or uncertain availability of (or access to) nutritionally-adequate, safe and socially-acceptable foods (Core Indicators of Nutritional State for Difficult-to-Sample Populations, 1990), has been associated with increased hospitalization, anemia, anxiety and depression, and lower academic performance (Bhattacharya et al., 2004, Black et al., 2012, Cook et al., 2004, Cook et al., 2013, Hendrickson et al., 2010, Ke and Ford-Jones, 2015, Metallinos-Katsaras et al., 2016, Olson, 1999, Skalicky et al., 2006;). A recent United Nations survey of European countries estimated that in the UK over 10% of people aged 15 years and above experienced food insecurity (Too Poor to Eat, Food Insecurity in the UK). For just under 5% of people surveyed, food insecurity was severe, meaning they sometimes went without eating for an entire day because they did not have enough money to purchase food (Too Poor to Eat, Food Insecurity in the UK). Paradoxically, food insecurity may be a determinant of obesity (Dinour et al., 2007; Nackers and Appelhans, 2013).

The majority of evidence, largely from the United States (US) and Canada, has linked food insecurity with obesity and weight gain in adults. But the experience of food insecurity by children, particularly very young children, has also been associated with obesity in both cross-sectional and longitudinal studies (Dinour et al., 2007, Eisenmann et al., 2011, Metallinos-Katsaras et al., 2009, Metallinos-Katsaras et al., 2012, Speirs and Fiese, 2016). A recent study of children aged 2–5 years found that more than 25% of food insecure children were overweight or obese, which is higher than the overall average proportion of overweight and obesity in children across the US (Speirs and Fiese, 2016). Several explanations have been put forward to reconcile the apparent paradox of obesity existing within food-insecure families, including psychosocial stress and the reliance on energy-dense, nutrient poor foods, which are low-cost and affordable for those experiencing financial hardship (Drewnowski and Specter, 2004), and especially palatable and acceptable to children (Daniel, 2016).

Taken together, child obesity and food insecurity constitute complex and interrelated challenges to public health. While obesity in the population is closely tracked, the study of food insecurity in the UK population has been largely neglected. Considering the social and health consequences of food insecurity among children (Cook et al., 2004, Cook et al., 2013, Fotso et al., 2012, Hendrickson et al., 2010, Metallinos-Katsaras et al., 2016, Pilgrim et al., 2012;) more information is urgently needed on the scope and extent of the problem in the UK. Moreover, little to no information is available on the association between food insecurity and obesity in the UK.

Nurseries may provide important insight into the state of food insecurity among children. The majority of children under the age of five years spend time in out-of-home child care, and the amount of time in care increases as children age (Childcare and early years providers survey 2013, Kamerman, 2000). The number of children in early years settings in England has more than doubled in the past decade, with 796,500 in care in 2013 (Childcare and early years providers survey 2013, Kamerman, 2000). The purpose of this study was to assess manager perceptions of both food security and obesity in children attending early years settings, assessing differences by area socioeconomic status across England. We hypothesized that perceptions of both food insecurity and obesity in children would be highest in the most deprived areas of England.

## 2 Methods

### 2.1 Sample

We administered a survey by post to a stratified random (cross-sectional) sample of 2000 nurseries (determined by available funds) in England from November 2012 to September 2013. Details of the survey protocol are available elsewhere (Neelon et al., 2015). Briefly, we obtained the names of all 28,091 registered nurseries in England from Ofsted, the agency responsible for regulating early years programs in England. Nurseries include any group or organization that provides care for children more than six days a year, and for at least two hours a day on non-domestic premises. To be included in the study, Ofsted regulated nurseries needed to provide at least one meal or snack to children in care daily, and care for children under six years of age on a regular basis (e.g., not simply during holidays or after school hours). Programs were excluded if they were a sports club or camp for children, served children with special dietary needs only, or cared for children over six years of age exclusively. We designed the survey to be completed by the manager in about 20 min, without review of any nursery documents or input from parents. We did ask managers to seek input from other child care providers in their nurseries as needed. We provided nursery managers with a £15 voucher after they completed the survey. The survey included a letter to the manager stating that completion of the survey constituted consent to participate in the study. All study procedures were approved by the University of Cambridge Psychology Research Ethics Committee.

Using the list provided by Ofsted, we geocoded all 28,091 nursery addresses at the postcode level, using a geographic information system (GIS) (ArcGIS 10, ESRI Inc., Redlands, CA) and used the geocoded addresses to classify nurseries within lower super output areas (LSOAs), which are small administrative boundaries containing about 1500 individuals. Next, we stratified nurseries based on LSOA tertile (low, middle, high) using the index of multiple deprivation (IMD) 2010 scores (The English Indices of Deprivation, 2011), the most recent scores available at the time the nursery survey was administered. The IMD measures relative deprivation and is published by the Department for Communities and Local Government in England. The IMD is updated every three to four years, is a compound measure of material deprivation, and includes aspects of unemployment, housing prices, income, crime, and education levels, within LSOAs. As noted previously (Neelon et al., 2015), we oversampled nurseries in the most deprived areas (the highest tertile of IMD) to reduce selection bias, expecting a lower response rate from nurseries in these areas, sending surveys to 500 in the low, 500 in the middle, and 1000 in the high IMD tertile.

### 2.2 Survey

The purpose of the survey was to assess current practices related to food access and availability, behaviors related to feeding children in care, and activities to promote healthy eating among children in a sample of nurseries across England. We developed the survey using previous instruments designed to assess nutrition- and obesity-related practices within child care programs in the US (Benjamin et al., 2007; Ward et al., 2008; Whitaker et al., 2009), modifying the questions and response options as needed for use in England. To assess manager perceptions of obesity in children attending their nursery, the survey asked “How much of a problem is obesity in your program among children?”, with five possible responses: “not a problem”, “small problem”, “moderate problem”, “large problem”, and “very large problem”. For analyses, we dichotomized nurseries into managers who perceived obesity among their children as “not a problem”, relative to those who reported obesity among children in their care in any other way.

Questions regarding food security were based on those used in a survey previously conducted to assess Head Start program practices (Whitaker et al., 2009); an early years program within the US Department of Health and Human Services that provides care and education to low-income children and their families. Managers were asked “Do you or your staff feel that some children in your program do not get enough food to eat at home?”, with response options including “never or rarely”, “sometimes”, and “often”. For analyses we dichotomized responses as “never or rarely” versus “sometimes” and “often”; we interpreted the latter as evidence of food insecurity within the nursery. While food security among children is typically measured by asking the parent or the primary caregiver, we were interested in assessing the manager's perception of food insecurity and how the nursery responded to their concern. The follow-up question asked “What do you do when you or your staff are concerned that children are not getting enough food to eat at home?”. Response options included “feed more on Mondays and Fridays to make up for weekends”, “keep additional food on hand to feed the child during the day”, “give food to the family to take home for the child to eat”, “refer the family to Sure Start Children's Centers, social services, or a charity”, and “talk to parents”, and managers were instructed to mark all that apply.

We also asked a number of questions about how the nursery was owned and operated (privately owned versus part of a corporation or chain), the number of children within the nursery, and the number of years the nursery has been in operation. Additionally, we asked nursery managers to report their age, sex, highest education (GCSEs, A-levels, National Vocational Qualifications, two-year diploma, degree, or higher degree), years employed by their current nursery, and years of experience in child care.

### 2.3 Global statistical analysis

We conducted binary logistic regression analyses, examining the odds of a manager perceiving only obesity as a problem among children, perceiving only food insecurity as a problem, or perceiving both as a problem, for each tertile of deprivation relative to those least deprived. Models were adjusted for the total number of children enrolled in each nursery, whether a nursery was privately owned or part of a corporation or chain, and manager level of education (dichotomized as less than a two-year diploma versus a two-year diploma or higher). After adjustment, total number of children made no meaningful difference to the relationship between deprivation tertile and the outcome, and was not significant it its own right, and so was removed from the final model. Additionally, as a sensitivity analysis, we performed multiple imputation with fully conditional specification, with 10 imputations, and which included the outcome and all covariates. Regression results are presented as odds ratios (OR) with 95% confidence intervals (CI), and two-sided *p*-values. Analyses were conducted using SAS version 9.4 (SAS Institute, Cary, North Carolina, US).

### 2.4 Local statistical analysis

We conducted a logistic geographically weighted regression (GWR) analysis, examining the odds of a manager perceiving both obesity and food insecurity as problems, for each tertile of deprivation relative to those least deprived. Models were adjusted as per our global analyses. GWR predicts nonstationarity in relationships by producing a ‘local’ parameter estimate for each geographic location (in this case, each nursery) based on a proximal subset of the global data (Matthews and Yang, 2012). The GWR equation is as follows:

$$\gamma_{i\;}\left(u\right)=\;\beta_{1}\left(u\right)x_{1}+\;\beta_{2}\left(u\right)x_{2}+\ldots +\;\beta_{n}\left(u\right)x_{n}$$

where *y_i_* is the dependent variable at location *u, β*_1_(*u*) is an estimate of the regression co-efficient for *x*_1_ as the product of a distance decay function surrounding location *u*, and to which this parameter is unique (Geographically Weighted Regression: White Paper). To model distance decay, our analyses used an adaptive bi-square geographic kernel, with bandwidth determined by akaike information criterion (AIC) minimization to account for the sparseness of nursery locations in some parts of England. Logistic GWR results are presented as mapped ORs with t-values ≥±1.96 indicating statistical significance. Relationships were deemed to exhibit nonstationarity across the study area where the interquartile range of the local estimates for deprivation was more than double the standard error of the global estimate (Fraser et al., 2012). Goodness of model fit is compared between global and local models using corrected AIC values, where a lower value by more than two points is indicative of better model fit (GWR4.09 User Manual). Analyses were conducted using GWR4.0 (National Centre for Geocomputation, National University of Ireland Maynooth, Ireland), with ORs mapped using a GIS (ArcGIS 10, ESRI Inc., Redlands, CA).

## 3 Results

### 3.1 Demographics

Of the 2000 nurseries mailed a survey, 202 (10%) were no longer in operation. We excluded an additional 230 (11%) because they were not an early years setting, did not care for children regularly, or did not provide any food to children. Of the remaining 1568 nurseries, 851 (54%) returned a survey. Of those nurseries, four were missing information on tertile of deprivation, 56 did not respond to the question on manager education level, and 96 did not respond to the question on nursery ownership, leaving a final sample for the adjusted analysis of 707. Thus, we included only nurseries with complete exposure, outcome, and covariate data in our final sample for analysis.

**Fig. 1:**
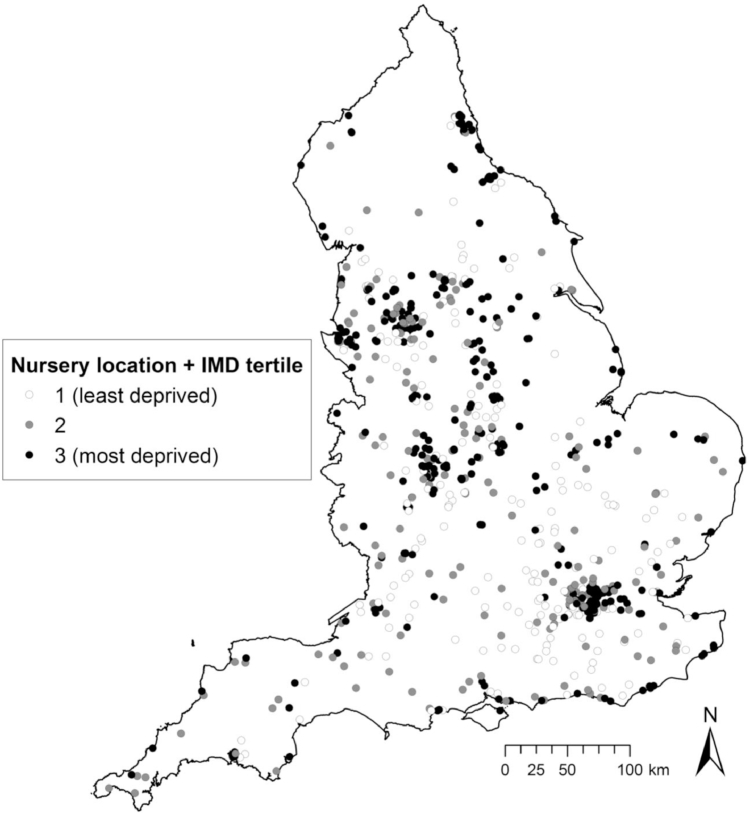
Locations of participating nurseries in the analytic sample throughout England (n = 707), and their distribution across lower super output area index of multiple deprivation 2010 tertiles. © Crown Copyright/database right 2017, an Ordnance Survey/EDINA supplied service.

Responding nurseries were located throughout England (Fig. 1), and relatively equally distributed across deprivation tertiles, with 56% in the least deprived, 56% in the middle deprived, and 52% in the most deprived LSOAs (Neelon et al., 2015). Among the 707 nurseries in our analysis, most nursery managers (96.6%) were women, and had a mean (SD) of 16.9 (9.2) years of experience working in nurseries (Table 1). The mean age of managers was 42.4 (11.0) years. Among managers, 73 (10.3%) perceived only obesity as a problem among children, 188 (26.6%) perceived only food insecurity as a problem, 121 (17.1%) perceived both as a problem, and 325 (46.0%) perceived neither as a problem. Perceptions of food security being a problem, and of both food security and obesity being problems, were patterned by area-level deprivation. Nursery managers in the most deprived areas were more likely to perceive problems with food insecurity (31.7%) and food security and obesity (27.6%), than those in the least deprived areas (20.5% and 6.8%, respectively).

**Table 1:** Demographic characteristics of nurseries and managers in the Nutrition in Nurseries survey (n = 707) by area-level deprivation.

	Total sample (n = 707)	Least deprived (n = 190)	Middle deprived (n = 195)	Most deprived (n = 322)
**Nursery characteristics**	Number (%)			
Obesity and food security problems				
Neither problem	325 (46.0)	119 (62.6)	105 (53.8)	101 (31.4)
Obesity problem only	73 (10.3)	19 (10.0)	24 (12.3)	30 (9.3)
Food security problem only	188 (26.6)	39 (20.5)	47 (24.1)	102 (31.7)
Obesity and food security problem	121 (17.1)	13 (6.8)	19 (9.7)	89 (27.6)
Nursery type				
Private owner	169 (23.9)	37 (19.5)	42 (21.5)	90 (28.0)
Part of corporation or chain	538 (76.1)	153 (80.5)	153 (78.5)	232 (72.0)
	Mean (SD)			
Years in operation	16.5 (11.8)	18.6 (12.7)	18.0 (11.8)	14.5 (11.0)
Cost of care per month, £				
For infants	516.8 (356.6)	617.3 (356.0)	564.9 (410.4)	460.1 (321.7)
For toddlers	478.0 (341.8)	542.5 (355.7)	470.1 (377.6)	455.6 (314.2)
For preschoolers	392.9 (316.1)	438.8 (325.9)	374.1 (335.2)	382.1 (299.8)
**Manager characteristics**	Number (%)			
Sex, female	673 (96.6)	184 (97.4)	184 (96.3)	305 (96.2)
Education				
Less than 2-year degree	293 (41.4)	78 (41.1)	92 (47.2)	123 (38.2)
2-year degree or higher	414 (58.6)	112 (58.9)	103 (52.8)	199 (61.8)
	Mean (SD)			
Age, years	42.4 (11.0)	42.9 (10.2)	42.2 (11.6)	42.3 (11.2)
Years worked in child care	16.9 (9.2)	16.5 (9.2)	17.0 (9.0)	17.1 (9.3)

### 3.2 Global associations between obesity and food insecurity as problems, and area deprivation

After adjusting for whether a nursery was privately owned and manager level of education, we found that a nursery being in the highest tertile of deprivation was associated with a 1.89 (95% CI 1.00, 3.57; *p* = 0.049) times greater odds of perceiving only obesity as a problem; a 3.06 (95% CI 1.94, 4.84; *p* < 0.001) times greater odds of perceiving only food insecurity as a problem; and a 8.39 (95% CI 4.36, 16.15; *p* < 0.001) times greater odds of perceiving both as a problem, all relative to those in the lowest tertile of deprivation (Table 2). The overall F-test *p*-value for the tertile effect was <0.001. We did not observe a significant difference comparing the middle to the lowest tertile of deprivation for any of our outcomes. In the fully adjusted multiple imputation model, the estimates changed only slightly but did not change in terms of significance (data not shown).

**Table 2:** Associations of area-level deprivation and each of obesity, food insecurity, or both, estimated using individual binary logistic regression models.

	OR (95% CI)	P-value
**Obesity problem only** (n = 398)		
Low deprivation	Ref.	–
Middle deprivation	1.47 (0.76, 2.84)	0.26
High deprivation	1.89 (1.00, 3.57)	0.049
**Food insecurity problem only** (n = 513)		
Low deprivation	Ref.	–
Middle deprivation	1.40 (0.85, 2.32)	0.19
High deprivation	3.06 (1.94, 4.84)	<0.001
**Both obesity and food security problems** (n = 446)		
Low deprivation	Ref.	–
Middle deprivation	1.73 (0.81, 3.73)	0.16
High deprivation	8.39 (4.36, 16.15)	<0.001

^a^*Note*: Adjusted for whether a nursery was privately owned or part of a corporation or chain and manager level of education.

### 3.3 Local associations between obesity and food insecurity as problems, and area deprivation

**Fig. 2:**
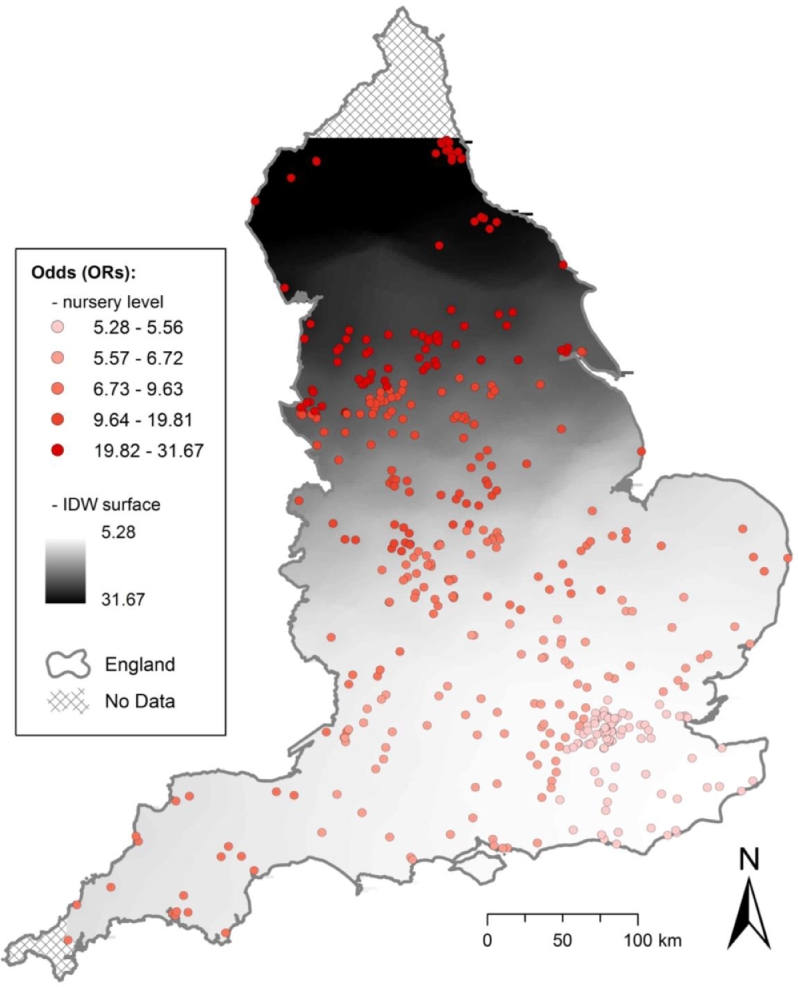
Association of area-level deprivation (showing quintiles of local odds ratios for highest deprivation tertile only^a^) and both obesity and food insecurity reported as problems^b^, estimated using logistic geographically weighted regression, using data from the Nutrition in Nurseries survey (n = 446). Odds ratios attributed to each nursery location based on local analyses of a proximal subset of the global data. © Crown Copyright/database right 2017, an Ordnance Survey/EDINA supplied service. ^a^ All odds ratios are significant, with t-values ≥1.96. ^b^ Adjusted for whether a nursery was privately owned or part of a corporate chain and manager level of education.

Our adjusted logistic GWR model revealed marked differences over space in the relationship between area-level deprivation and nursery manager perception of both obesity and food insecurity as problems. Fig. 2 shows local ORs (quintiles) by nursery location, as well as an inverse distance weighted surface representing these odds. All ORs demonstrated a positive relationship between area-level deprivation and odds of perceiving both obesity and food insecurity as a problem, and were significant (t-values ≥ 1.96). The observed relationship is stronger in the North of England, and relatively less strong in the South, South-East, and in Greater London. In one area of the North West, we found that a nursery being in the highest tertile of deprivation was associated with 31.67 times greater odds of perceiving both obesity and food insecurity as a problem, compared with a nursery being in the lowest tertile of deprivation. The local model showed better fit than the global model, with marked differences in corrected AIC values (global, 424.7; local, 421.9). These differences exhibited nonstationarity, with the interquartile range for local model estimates (1.21) more than double the standard error of the global model estimates (0.34).

## 4 Discussion

In this study of nurseries in England, we found that area deprivation was associated with manager perceptions of obesity, food insecurity, and in particular of both obesity and food insecurity combined. There is some evidence that food insecurity and obesity may co-exist within communities, families, and even within individuals. The underlying mechanism linking food insecurity with obesity include food consumption cycling—overconsumption in times of food abundance to account for anticipated scarcity later (Heitmann et al., 2012, Wells, 2006)—and intake of calorie-dense, nutrient-poor foods, which are economical, palatable and filling but can promote passive overconsumption (Cornell et al., 1989, Erlanson-Albertsson, 2005). While the precise pathways linking food insecurity and obesity in young children are not known, studies suggest that unhealthy dietary intake and inappropriate feeding behaviors may play a role (Bronte-Tinkew et al., 2007, Feinberg et al., 2008, Gross et al., 2012, Gross et al., 2014, Kaiser et al., 2002). Bronte-Tinkew et al. (2007) observed an indirect association between household food insecurity during infancy and obesity at two years, working primarily through parenting and feeding practices in a sample in the US. Feinberg et al. (2008), studied 278 mothers and children living in urban US environments and found that food insecure mothers were more likely to give their preschool- and school-age children high-energy supplements and appetite stimulants. In our study, we found that over one fifth of managers reported feeding children more on Mondays and Fridays or keeping additional food on hand at the nursery for hungry children, and these practices were more prevalent in the most deprived areas. These behaviors could in fact contribute to obesity, if managers are feeding children more food whilst in care to compensate for inadequate calories at home.

We also showed geographic variation in the magnitude of the association between area deprivation and manager perception of both obesity and food insecurity, using a GWR method. There is some precedent for the use of GWR in recent obesity research (Black, 2014, Fraser et al., 2012), however to our knowledge, GWR has not yet been utilized to study the relationship between area deprivation and nursery characteristics in any setting. While the relationships observed were aligned with those from global models, perceptions of nursery managers of both obesity and food insecurity being problems were more closely tied to area deprivation in particular hotspots, for example, in the North of England. The spatial variation in this relationship was masked by the global models, with GWR models exhibiting marked spatial nonstationarity and providing better overall model fit. The diversity in magnitude of associations revealed should also help to drive the research agenda in so far as they necessitate a deeper, region-specific understanding of other contributors to nursery level obesity and food insecurity problems.

This study has some limitations. Our assessment of obesity was based on manager perception, and not actual measures of children's obesity status. While measured height and weight would have provided more precise information, the purpose of the study was to collect data on a large sample of nurseries across England to inform and guide future research. Findings from this survey will help identify geographic areas of focus for additional research that should include more accurate measures of obesity in children. Similarly, our measure of food insecurity was not based on parent report to reflect food insecurity within the household, which is the standard approach used to identify food insecurity in children. Food security status may not be readily identifiable in young children and manager perception may not reflect actual food insecurity. However, this alleviates some of the social stigma and thus social desirability or response bias associated with parent report of household food insecurity. However, it is possible that perceived food insecurity may underestimate actual food insecurity, if outward signs are not readily apparent in children.

Additionally, we asked managers to seek input from other child care providers in the nursery, as needed, to complete the survey. Some managers may have sought assistance from child care providers, but others may not have requested this input. While managers likely have a better grasp of practices across the entire nursery, child care providers within the classroom may be better positioned to assess food insecurity due to their proximity to and interactions with children. Geographically weighted regression is also not without limitations, which include issues related to multicollinearity, kernel bandwidth selection and study area edge effects. This study is also limited in its generalizability and thus external validity by the 54% response rate. However, the nursery managers who responded were distributed across England, and response rates were largely similar by area deprivation. We anticipated a lower response rate from nurseries in the most deprived areas, and to reduce potential selection bias we oversampled those nurseries to ensure adequate representation. However, response rates across tertiles of deprivation were similar, so this may not have been necessary.

Finally, our study reflects a snapshot of nursery conditions and practices in 2012–13 and did not capture important trends in food insecurity occurring since this time. Although high food price inflation in the UK since 2008 began to moderate in 2012 (Department for Environment FaRA: Family Food 2015, 2017), household food insecurity is likely to be a large and growing concern. The prevalence of food insecurity is not routinely estimated in the UK, but a report using data from the United Nations estimated that 8.4 million UK residents were food insecure in 2014 (Taylor and R, 2016). Moreover, the use of food banks in the UK, one indicator of food insecurity, increased every year between 2008 and 2016–17, with more than a three-fold increase between 2012–13 and 2016–17 (Butler, 2017). More direct monitoring of food insecurity is needed in England and throughout the UK, particularly for households that include children.

At present, mandatory nutrition requirements for food in nurseries are minimal, stipulating only that food and drink served should be ‘healthy, balanced and nutritious’ (Office for Standards in Education Children's Services and Skills, 2013). However, recent national reports and papers have called for enhanced standards in nurseries and other child care settings (Buttivant and Knai, 2012, Children's Food Trust, 2012, Department for Education 2012). The most comprehensive nutrition standards for nurseries, developed in 2010, are voluntary (Department for Children SaFSFT, 2010), but some have called for a clear and unambiguous definition of “healthy, balanced and nutritious” in the mandatory regulations to help promote healthy eating and prevent obesity (Children's Food Trust, 2012).

## 5 Conclusions

In this study assessing food insecurity and obesity in young children, we found that area deprivation was associated with manager perceptions of both obesity and food insecurity, but the relationship was especially pronounced for food insecurity. Recent national efforts call for improved nutrition in early years settings to help prevent obesity in young children (Childhood Obesity: A Plan for Action). It is also important to consider issues of food insecurity, and the potential for food insecurity and obesity to coexists in young children, when responding to these calls.
